# Dyshidrosiform Bullous Pemphigoid

**DOI:** 10.3390/medicina57040398

**Published:** 2021-04-20

**Authors:** Philip R. Cohen

**Affiliations:** 1 San Diego Family Dermatology, National City, CA 91950, USA; mitehead@gmail.com; 2 College of Osteopathic Medicine, Touro University California, Vallejo, CA 94589, USA; 3 Scripps Family Medicine Residency, Scripps Mercy Hospital Chula Vista, Chula Vista, CA 91910, USA; 4 Family Medicine Residency, Family Health Centers of San Diego, San Diego, CA 92122, USA; 5 Davis Medical Center, Department of Dermatology, University of California Davis, Sacramento, CA 95814, USA

**Keywords:** blister, bullous, corticosteroid, dyshidrosiform, dyshidrosis, dyshidrotic, elderly, pemphigoid, pompholyx, vesicle

## Abstract

Dyshidrosiform bullous pemphigoid is a variant of bullous pemphigoid. At least 84 patients with dyshidrosiform bullous pemphigoid have been described. Dyshidrosiform bullous pemphigoid usually presents with pruritic blisters in elderly individuals; the hemorrhagic or purpuric lesions on the palms and soles can be the only manifestation of the disease. However, bullae may concurrently or subsequently appear on other areas of the patient’s body. Patients typically improve after the diagnosis is established and treatment is initiated. The mainstay of therapy is systemic corticosteroids, with or without topical corticosteroids, and systemic dapsone or immunosuppressants. Drug-related or nickel-induced dyshidrosiform bullous pemphigoid improves after stopping the associated agent; however, systemic therapy has also been required to achieve resolution of the blisters. Similar to classic bullous pemphigoid, neurologic conditions and psychiatric disorders have been observed in dyshidrosiform bullous pemphigoid patients. The new onset of recurrent or persistent blisters on the palms, soles, or both of an elderly individual should prompt the clinician to consider the diagnosis of dyshidrosiform bullous pemphigoid.

## 1. Introduction

Bullous pemphigoid is an autoimmune blistering condition that typically presents in older individuals. Pruritic tense subepidermal blisters usually develop in flexural areas that are proximally located, such as the axilla and the groin. The blisters can be localized or widespread [1,2,3,4,5,6,7,8,9,10,11,12,13,14,15,16,17,18,19,20,21,22,23,24,25,26,27,28,29,30,31,32,33,34,35,36,37,38,39,40,41,42,43,44,45,46,47,48].

Dyshidrosiform bullous pemphigoid is an unusual clinical variants of bullous pemphigoid. A common clinical scenario is the recurrent or persistent development of a pompholyx-like dermatosis—often hemorrhagic or purpuric—on the palms or soles or both of an elderly person. In these individuals, the palmar and plantar blisters either initially and only appear at these sites or initially appear on the hands and feet and subsequently develop at other sites or initially, and concurrently appear on the distal extremities and also at other sites. The features of patients with dyshidrosiform bullous pemphigoid are reviewed [4,5,6,7,8,9,10,11,12,13,14,15,16,17,18,19,20,21,22,23,24,25,26,27,28,29,30,31,32,33,34,35,36,37,38,39,40].

## 2. Inceptive Report

Levine et al. provided the initially description of dyshidrosiform bullous pemphigoid in the March 1979 issue of Archives of Dermatology. In addition to their patient’s rare oral and truncal lesions, the authors considered the 72-year-old man’s clinical presentation to be indistinguishable from plantar dyshidrosiform dermatitis [4].

He presented with a vesicular eruption of the hands; then, two weeks after treatment with tar soaks and topical corticosteroid application it resolved. However, within two weeks, his feet developed large tense bullae; after two weeks of daily oral prednisone (40 mg), the bullae had cleared [4].

Periodically blisters continued to appear on his feet; one of the blistering episodes was accompanied by truncal lesions and a pharyngeal erosion. Therapies—such as oral erythromycin and intramuscular corticosteroid—were successful; however, the clinical improvement was not sustained. A potassium hydroxide preparation of a skin scaping from his from foot demonstrated hyphae; during treatment with griseofulvin, many new bullae appeared [4].

A skin biopsy of his left plantar foot was performed. The findings of both the light microscopy and direct immunofluorescence studies of the tissue specimen established the diagnosis of bullous pemphigoid. Indirect immunofluorescence studies of the patient’s serum also confirmed the diagnosis [4].

The bullae on his feet all resolved within seven days after starting sulfapyradine (500 mg thrice daily); however, he could not tolerate the medicine and it was stopped one week later. New lesions again appeared; they promptly cleared within five days after starting dapsone (50 mg twice daily). His treatment was stopped after four months and no new blisters appeared during the next three months [4].

## 3. Materials and Methods

A retrospective review of the published literature on dyshidrosiform bullous pemphigoid was performed. The Pubmed database was used. The following key words were searched: blister, bullous, corticosteroid, dyshidrosiform, dyshidrosis, dyshidrotic, elderly, pemphigoid, pompholyx, and vesicle. Relevant articles and the references cited in these articles were evaluated for inclusion.

Dyshidrosiform bullous pemphigoid has been described in at least 84 patients. A single patient was reported in 37 papers. Several of the articles included more than one individual: either two patients (two papers), three patients (five papers), four patients (one paper), nine patients (one paper), or 20 patients—one of whom was previously described—(one paper) [4,5,6,7,8,9,10,11,12,13,14,15,16,17,18,19,20,21,22,23,24,25,26,27,28,29,30,31,32,33,34,35,36,37,38,39,40].

## 4. Incidence

Three studies of bullous pemphigoid patients include information regarding the subset of individuals with dyshidrosiform bullous pemphigoid [17,18,19]. One study only identified three patients with dyshidrosiform bullous pemphigoid in 86 individuals with bullous pemphigoid. Hence, individuals with dyshidrosiform bullous pemphigoid merely represented 3.5% of the bullous pemphigoid patients treated at the largest teaching hospital in Taiwan from 1977 to 1994 [19].

A second study, evaluating palmoplantar involvement in autoimmune blistering disorders, included 71 bullous pemphigoid patients from the Oxford and St. John’s Hospital, London bullous disease clinics. The researchers observed the incidence of dyshidrosiform bullous pemphigoid to be 28% (20 individuals). They also noted blisters on the palms and soles in 12% (three of 26 individuals) of linear IgA disease patients and 100% (two of two individuals) of herpes gestationis patients; none of the patients (zero of 35 individuals) with chronic bullous disease of childhood had blisters on their hands or feet [18].

The third study was evaluating clinical variations and the duration of prodromal symptoms in patients with bullous pemphigoid. The investigation included 20 bullous pemphigoid patients seen by the staff from the Department of Dermatology at the Sodersjukhuset Hospital in Stockholm, Sweden, during a period of three years. The researchers noted that 45% (nine individuals) of the patients had dyshidrosiform bullous pemphigoid [17].

In summary, the incidence of dyshidrosiform bullous pemphigoid remains to be established. Retrospective studies of bullous pemphigoid patients attempting to identify the number of dyshidrosiform bullous pemphigoid patients demonstrate the incidence of this variant of bullous pemphigoid to range from 3.5 to 45% (median, 28%). When the data from each of the three studies is combined, 18% (32 of 177 individuals) of the bullous pemphigoid patients had dyshidrosiform bullous pemphigoid [17,18,19].

## 5. Epidemiology

Epidemiology information was available for 48 of the 83 dyshidrosiform bullous pemphigoid patients. The individuals with dyshidrosiform bullous pemphigoid were either adult patients (44 individuals) or pediatric patients (four individuals: three boys and one girl). Most of the adult patients (89%, 39 of 44 individuals) were over 60 years of age at the time of diagnosis [4,5,6,7,8,9,10,11,12,13,14,15,16,17,18,19,20,21,22,23,24,25,26,27,28,29,30,31,32,33,34,35,36,37,38,39,40].

In the group of patients with juvenile bullous pemphigoid, three had childhood bullous pemphigoid (with an onset age between one year and 18 years) and one had infantile bullous pemphigoid (with an onset age of 12 months or younger) [30,34]. In a comprehensive literature review of infantile and childhood bullous pemphigoid patients published in 2006 and earlier, the investigators noted the increased prevalence of blisters on the palms and soles; specifically, palmoplantar involvement of bullous pemphigoid was observed in 79% (31 of 39 patients) of infants and 17% (6 of 36 patients) of children [43]. Hence, it might be reasonable to only consider the diagnosis of dyshidrosiform bullous pemphigoid in bullous pemphigoid patients with blisters on the palms and soles whose disease onset occurs after the age of 19 years during adulthood.

Dyshidrosiform bullous pemphigoid in adults was equally common in men (22 patients) and women (22 patients). The onset age in men ranged from 20 years to 92 years (median, 75 years). The onset age in women ranged from 23 years to 94 years (median, 77 years). Overall, for all adult dyshidrosiform bullous pemphigoid patients, the onset age ranged from 20 years to 94 years (median, 76 years); for the remainder of this paper, dyshidrosiform bullous pemphigoid patients refer to individuals with adult onset [4,5,6,7,8,9,10,11,12,13,14,15,16,17,18,19,20,21,22,23,24,25,26,27,28,29,30,31,32,33,34,35,36,37,38,39,40]. 

## 6. Clinical Presentation

Dyshidrosiform bullous pemphigoid is characterized by the appearance of blisters on the palms, the soles, or both in a patient in whom the diagnosis of bullous pemphigoid has been or is subsequently established. Most of patients had blisters on both their palms and soles (24 of 36 individuals, 67%). However, nearly a third of the patients presented with blisters either only on their palms (one of 36 individuals, 3%) or only on their soles (11 of 36 individuals, 30%) [4,5,6,7,8,9,10,11,12,13,14,15,16,17,18,19,20,21,22,23,24,25,26,27,28,29,30,31,32,33,34,35,36,37,38,39,40].

The acral-located blisters can present in one of three clinical scenarios. The first scenario occurs when the blisters can be localized only to the palms and soles with no additional bullous pemphigoid lesions; this was observed in 28% (10 of 36 individuals) of the dyshidrosiform bullous pemphigoid patients. In these individuals, the diagnosis of this bullous pemphigoid subtype is often delayed since the patient is often mistakenly interpreted to have pompholyx and repeatedly treated for recurrent or persistent dermatitis.

**Figure 1:**
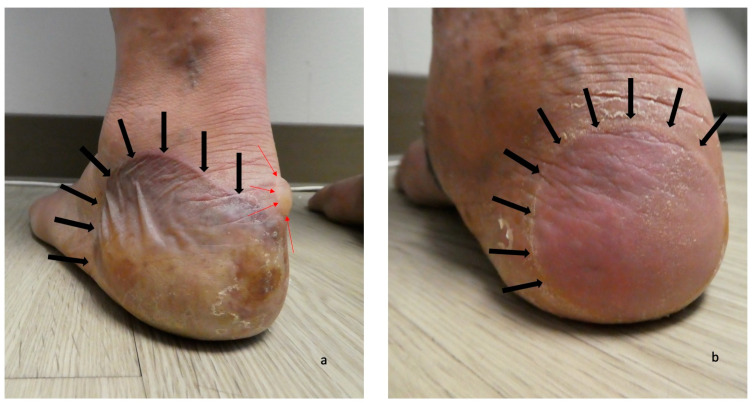
Dyshidrosiform bullous pemphigoid presenting as plantar blisters. A tender large flaccid hemorrhagic blister (**a**) and a flattened blister (**b**) on the posterior and plantar left heel (**a**) and right heel (**b**) of a 61-year-old man are the initial clinical manifestations of dyshidrosiform bullous pemphigoid. The blisters are outlined by black arrows; a tense, clear fluid-containing blister (outlined in red arrows) is also present on the medial area of the left heel (**a**). The figure and legend were originally published in Cureus under the Creative Commons (CC-BY): https://creativecommons.org/licenses/ on 11 January 2020 (Cohen PR: Dyshidrosiform bullous pemphigoid: Case reports and review. Cureus. 2020 Jan 11:12(1):e6630) and are republished with permission.

**Figure 2:**
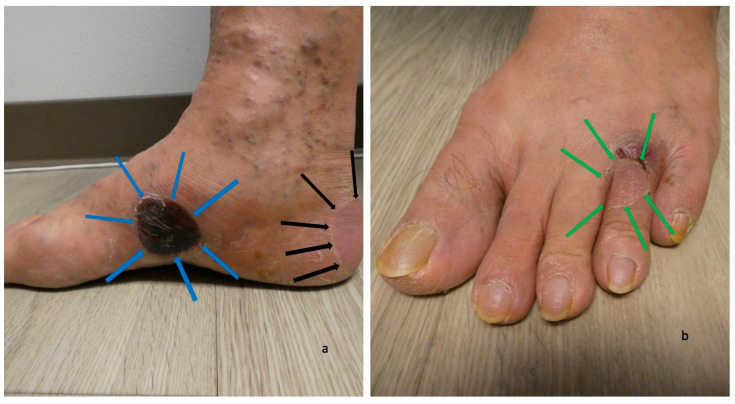
Blisters of dyshidrosiform bullous pemphigoid on the feet. A black-roofed and flattened blister on the instep (outlined in blue arrows) and another flattened blister that is primarily on the posterior heel and extends to the plantar foot (outlined in black arrows) on the medial foot (**a**) of a 61-year-old man. A deroofed blister is on the proximal fourth toe (outlined in green arrows) of the left dorsal foot (**b**). The figure and legend were originally published in Cureus under the Creative Commons (CC-BY): https://creativecommons.org/licenses/ on 11 January 2020 (Cohen PR: Dyshidrosiform bullous pemphigoid: Case reports and review. Cureus. 2020 Jan 11:12(1):e6630) and are republished with permission.

**Figure 3:**
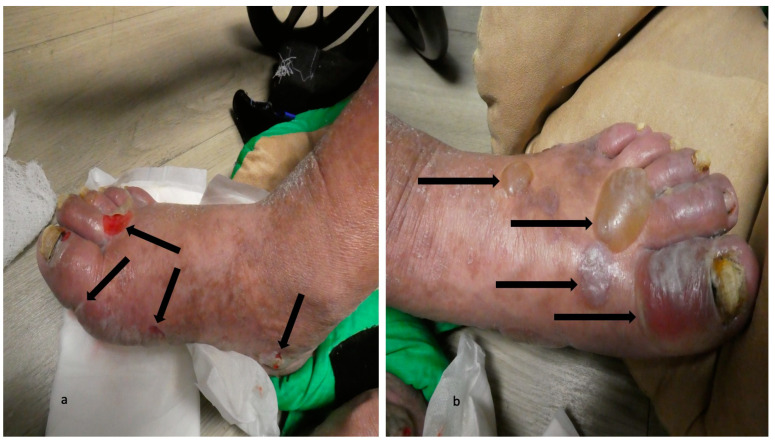
Dyshidrosiform bullous pemphigoid presenting as plantar blisters. Hemorrhagic and deroofed blisters (black arrows) on both the dorsal and lateral surface of the right foot (**a**) and left foot (**b**) of a 65-year-old man; some of the blisters extend to the soles. The figure and legend were originally published in Cureus under the Creative Commons (CC-BY): https://creativecommons.org/licenses/ on 11 January 2020 (Cohen PR: Dyshidrosiform bullous pemphigoid: Case reports and review. Cureus. 2020 Jan 11:12(1):e6630) and are republished with permission.

**Figure 4:**
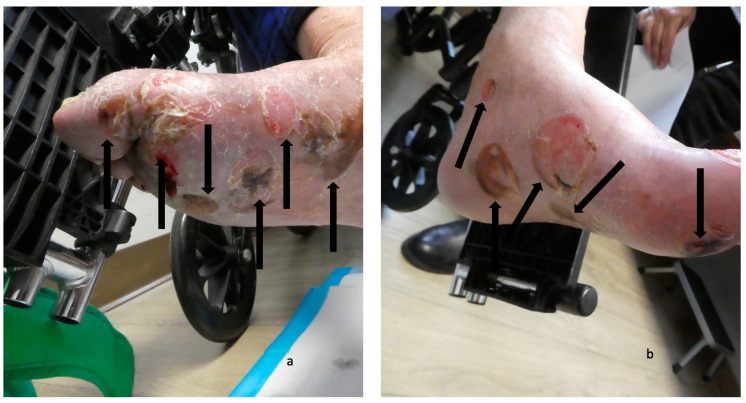
Blisters on the feet of a man with dyshidrosiform bullous pemphigoid. The plantar and medial surfaces of the right foot (**a**) and left foot (**b**) of a 65-year-old man with dyshidrosiform bullous pemphigoid show numerous flattened and deroofed blisters (black arrows). The figure and legend were originally published in Cureus under the Creative Commons (CC-BY): https://creativecommons.org/licenses/ on 11 January 2020 (Cohen PR: Dyshidrosiform bullous pemphigoid: Case reports and review. Cureus. 2020 Jan 11:12(1):e6630) and are republished with permission.

The second scenario involves the acral blisters preceding the development of blisters at other locations more typical of bullous pemphigoid lesions (Figure 1, Figure 2, Figure 3 and Figure 4). The duration of time between the onset of lesions on the palms and soles and new blisters on other body sites ranged from one week to seven months (median, seven weeks).

In the third scenario, the acral blisters and classically-distributed bulla of bullous pemphigoid appear at the same time. The latter two scenarios occurred in 72% (26 of 36 individuals) dyshidrosiform bullous pemphigoid patients.

Oral lesions were only described in five patients [6,11,16,27,33]; however, these kinds of lesions are usually associated with more superficial bullous disorders, such as pemphigus [46]. In addition to palmar and plantar blisters, four of the individuals also had lesions on other areas of their body [6,11,16,27]. In contrast, one dyshidrosiform bullous pemphigoid patient with oral lesions only had accompanying acral blisters [33].

Barth et al. were the initial investigators to highlight the association of hemorrhagic or purpuric blisters in dyshidrosiform bullous pemphigoid patients [6]. They suggested that ‘hemorrhagic pompholyx’ was a clinical sign of bullous pemphigoid [6]. Several researchers have subsequently emphasized the association of hemorrhagic blisters or purpuric lesion with dyshidrosiform bullous pemphigoid [7,16,22,23]. Indeed, hemorrhagic and purpura blisters were present in 86% of the patients (24 of 28 individuals), whose lesions were described in their individual case reports [4,5,6,7,8,9,10,11,12,13,14,15,16,17,18,19,20,21,22,23,24,25,26,27,28,29,30,31,32,33,34,35,36,37,38,39]. In contrast, only 5% patients (one of the 20 individuals) from a large series of dyshidrosiform bullous pemphigoid patients had hemorrhagic lesions [18].

Some of the dyshidrosiform bullous pemphigoid patients who had prodromal symptoms prior to the onset of their blisters. One man developed pruritic papules on his arms and upper back prior to the appearance of blisters on his palms [15]. In a study of 20 bullous pemphigoid patients, all nine of the dyshidrosiform bullous pemphigoid patients had prodomal symptoms either for greater than (five patients) or less than (four patients) three months. The symptoms included an eczematous eruption (three patients), a papular eruption (three patients), an eczematous and papular eruption (one patient), an intertriginous eruption (one patient), or an urticarial and papular eruption (one patient) [17].

## 7. Clinical Differential Diagnosis

The morphology of palmar and plantar dyshidrosiform bullous pemphigoid lesions mimics vesicular hand or foot dermatitis. In addition to dyshidrosis or pompholyx, the clinical differential diagnosis includes allergic and irritant contact dermatitis, chronic bullous disease of childhood, cutaneous T-cell lymphoma (vesicular palmoplantar variant), dermatophyte infection (bullous), epidermolysis bullosa aquisita, erythema multiforme, herpes gestationis, impetigo (bullous), lichen planus (bullous), linear IgA disease, scabies, and systemic contact dermatitis. Indeed, the possibility of an autoimmune bullous disease is often not initially considered in those individuals whose blisters only occur on their palms and soles [4,5,6,7,8,9,10,11,12,13,14,15,16,17,18,19,20,21,22,23,24,25,26,27,28,29,30,31,32,33,34,35,36,37,38,39,40].

## 8. Histopathology

All of the dyshidrosiform bullous pemphigoid patients had histopathologic confirmation of their bullous pemphigoid diagnosis. This included hematoxylin and eosin stained sections of a formalin-fixed skin lesion tissue specimen. The findings were similar to those typically observed in bullous pemphigoid; a subepidermal blister with or without an infiltrate of eosinophils in the dermis.

## 9. Immunofluorescence

Direct immunofluorescence was performed for most of the dyshidrosiform bullous pemphigoid patients. Similar to classical bullous pemphigoid, a continuous linear deposits of complement component 3 (C3) and immunoglobulin G (IgG) along the dermoepidermal junction and blister roof. Indirect immunofluorescence was also frequently performed; usually—but not always—the testing was also positive for bullous pemphigoid [11,16].

## 10. Western Blot Testing and Enzyme-Linked Immunosorbent Assay

Western blot testing and enzyme-linked immunosorbent assay for autoantibodies against bullous pemphigoid antigen one (BPAg1) or bullous pemphigoid antigen two (BPAg2) or both were not commonly performed for the earlier patients diagnosed with dyshidrosiform bullous pemphigoid. It is likely that the tests were either not available or not readily accessible. However, most of the dyshidrosiform bullous pemphigoid patients who have more recently been reported have had antibody testing to confirm the diagnosis [11,14,27,29,31,32,33].

## 11. Pathogenesis

The pathogenesis of dyshidrosiform bullous pemphigoid is postulated to be the same as that for classical bullous pemphigoid. The pathogenesis of bullous pemphigoid involves immunoglobulin G (IgG) autoantibodies targeting hemidesmosome proteins bullous pemphigoid 180 (BP180) and bullous pemphigoid 230 (BP230). BP230 (also known as BPAg1) is a 230 kilodalton (kDa) intracellular protein. BP180 (also known as BPAg2, or type XVII collagen, or Col17) is a transmembrane protein of 180 kDa. The extracellular non-collagenous 16A (NC16A) domain of BP180 contains immunodominant epitopes, and anti-NC16A IgG antibodies correlate with disease activity. Interestingly, immunoglobulin E (IgE) autoantibodies targeting pemphigoid antigens were demonstrated in serum and skin of bullous pemphigoid patients as well [1,2,3,40,41,42,47].

However, the predilection for dyshidrosiform bullous pemphigoid to have an affinity for the palms and soles remains to be determined. Hamm and Wozniak evaluated bullous pemphigoid antigen concentration in normal human skin in relationship to body area and age; they evaluated the sera with bullous pemphigoid antibody from two patients with 36 samples of normal skin (from six body areas from six cadavers). They not only found that the highest endpoint titers were from the oldest patients, but also that the greatest expression of bullous pemphigoid antigen was on plantar sites; the palms were not studied. Hence, perhaps this favored site of bullous pemphigoid antigen might account for the development of blisters on the soles (and palms) of patients with dyshidrosiform bullous pemphigoid [44].

Trigger factors have also been associated with either inducing or exacerbating bullous pemphigoid. Dyshidrosiform bullous pemphigoid was demonstrated to be induced by nickel in the diet in a 23-year-old woman with a six-year history of metal allergy (documented by positive patch testing to nickel sulfate) and a five-year history of a recurrent itchy versicular eruption of the hands and feet that was repeatedly treated with topical corticosteroids as pompholyx. Eventually, after the correct diagnosis of dyshidrosiform bullous pemphigoid was established, all of her lesions were able to be resolved and she was able to be maintained blister-free on a low-nickel diet. A single oral challenge of nickel resulted in new bullae within 24 hours, confirming the role of the metal as a trigger in the pathogenesis of her dyshidrosiform bullous pemphigoid [23].

Numerous drugs have been documented to trigger bullous pemphigoid. Lugovic-Mihic et al. proposed that baclofen induced dyshidrosiform bullous pemphigoid in their patient. However, there are several issues that may challenge whether the association between the drug and the onset of dyshidrosiform bullous pemphigoid is bon-a-fide or coincidental [33]. The issues include: (1) baclofen is not listed as a triggering agent in major reviews of drug-induced bullous pemphigoid, (2) the patient has a paraplegia secondary to ischemic transverse myelitis (and neurologic conditions can be associated with bullous pemphigoid), (3) the medication was started two years prior to the onset of dyshidrosiform bullous pemphigoid, and (4) after baclofen withdrawal, systemic corticosteroids in conjunction with an additional immunosuppressant (azathioprine) was still necessary to resolve the patient’s blisters [33,40,45].

Moro et al. presented clinical images of two patients with dipeptidylpeptidase 4 inhibitors (DPP-4i)—induced bullous pemphigoid; the second patient had dyshidrosiform bullous pemphigoid. Dipeptidylpeptidase 4 inhibitors—also referred to as gliptins—are oral hypoglycemic agents that have many associated potential adverse events including not only bullous pemphigoid, but also inflammatory bowel disease, multiple sclerosis, psoriasis, and thyroiditis. Bullous pemphigoid associated with dipeptidylpeptidase 4 inhibitors has presented as classical bullous pemphigoid, non-inflammatory localized bullous pemphigoid (such as dyshidrosiform bullous pemphigoid), and mucous membrane bullous pemphigoid. Some of the gliptins that have induced bullous pemphigoid include alogliptin, anagliptin, linagliptin, saxagliptin, sitagliptin, teneligliptin, and vildagliptin. The pathogenesis of dipeptidylpeptidase 4 inhibitors associated bullous pemphigoid remains to be determined; alteration in the correct cleavage of BPAg2 (resulting in modification of its antigenicity and function) by dipeptidylpeptidase 4 inhibitors-associated plasmin inhibition has been postulated as a potential mechanism [40].

## 12. Treatment

Treatment of dyshidrosiform bullous pemphigoid was described for 37 of the patients. Systemic corticosteroids were used in the management of most (31 individuals, 84%) of the dyshidrosiform bullous pemphigoid patients. The starting dose ranged from 10 to 80 mg daily (median, 30 mg daily) [4,5,6,7,8,9,10,11,12,13,14,15,16,17,18,19,20,21,22,23,24,25,26,27,28,29,30,31,32,33,34,35,36,37,38,39,40].

Other systemic medications were also used in the management of dyshidrosiform bullous pemphigoid patients. These included dapsone (eight individuals whose dose ranged from 50 to 200 mg daily, median 150 mg daily) [4,6,17,20,23,25,28,29], oral antibiotics (three individuals), such as erythromycin [22], doxycycline [16] or tetracycline [28], and nicotinamide (one individual) [28]. Topical corticosteroids (17 individuals) were also used as an adjunctive therapy [4,5,6,7,8,9,10,11,12,13,14,15,16,17,18,19,20,21,22,23,24,25,26,27,28,29,30,31,32,33,34,35,36,37,38,39,40].

Immunosuppressant drugs, either alone or as a corticosteroid agent, were also used to treat dyshidrosiform bullous pemphigoid. Azathioprine was used in four individuals; the dose ranged from 100 to 150 mg daily (median, 100 mg daily) [22,24,33,35]. Cyclophosphamide was used in one individual; the daily dose was 100 mg [22].

Withdrawal of a suspected triggering agent has also been suggested as a primary or an adjunctive measure in the treatment of dyshidrosiform bullous pemphigoid. A 23-year-old woman with patch test documented allergic contact dermatitis to nickel sulfate had a five-year history of palmar and plantar itchy vesicular eruption. After the diagnosis of dyshidrosiform bullous pemphigoid was established, she received dapsone 50 mg thrice daily with transitory improvement. Prednisolone 60 mg produced lesion resolution within 11 days; however, after tapering to 35 mg daily the lesions recurred. Initiation of a low-nickel diet was added and all of the blisters completely resolved within 15 days; bullae again recurred within 24 hours after a single oral challenge of 6 mg of metal nickel. The patient remained blister-free at 6 month follow-up on only a low-nickel diet [23].

Withdrawal of baclofen, in a 49-year-old man with paraplegia of the distal extremities secondary to ischemic transverse myelitis, was associated with regression and maintained clearance of dyshidrosiform bullous pemphigoid. The patient had been taking baclofen for two years prior to the onset of palmar and plantar blisters and oral lesions. His physicians considered baclofen to be the probable trigger for his dyshidrosiform bullous pemphigoid. Therefore, they discontinued the drug and introduced diazepam; systemic and topical corticosteroids and azathioprine were also started. All of his lesions cleared and the medications were tapered with no relapse at follow-up five years later [33].

Omalizumab, a monoclonal antibody targeting IgE, has been used therapeutically in several bullous pemphigoid patients, and may be a potential therapeutic agent for patients with dyshidrosiform bullous pemphigoid. A meta-analysis found complete responses in approximately 84% of patients, with decreased pruritus and blister development. These results imply an important role for IgE in the disease mechanism of bullous pemphigoid, and potentially in blister formation [48].

## 13. Follow-Up

Treatment resulted in improvement for nearly all of the dyshidrosiform bullous pemphigoid patients; however, when reported, many patients were still receiving therapy [4,5,6,7,8,9,10,11,12,13,14,15,16,17,18,19,20,21,22,23,24,25,26,27,28,29,30,31,32,33,34,35,36,37,38,39,40]. Typically, within 1 week to 1 month, resolution of lesions occurred. However, either during tapering or after stopping the systemic treatment, recurrent episodes of palm and sole lesions were not uncommon. Unrelated to dyshidrosiform bullous pemphigoid or its treatment, two of the patients died; one had a cardiac arrest and the other had respiratory failure [11,24].

## 14. Associated Disorders

An association between bullous pemphigoid and neurologic conditions has been recognized. Autoantibodies to epithelial PGAg1 (BPAg1-e) has been postulated to cross react against neuronal BPAg1 (BPAb1-n) which stabilizes the cytoskeleton of sensory nerves [1,2,3,28,40]. At least 10 of the dyshidrosiform bullous pemphigoid patients had a neurologic disorder: cerebrovascular accidents (two men and one woman) [6,11,15], Parkinsonism (two women and one man) [14,28,35], epilepsy (one man) [35], ischemic transverse myelitis and paraplegia (one man) [33], peripheral neuropathy (one woman) [16], and senile dementia (one woman) [11].

Psychiatric disorders were also observed in patients with dyshidrosiform bullous pemphigoid. One man had manic depression syndrome [35]. Similarly, one of the women with dyshidrosiform bullous pemphigoid suffered from depression [14]. Whether dyshidrosiform bullous pemphigoid patients have an increased incidence of neurologic and psychiatric conditions as compared to individuals with bullous pemphigoid who do not have dyshidrosiform-like lesions remains to be established.

## 15. Conclusions

Bullous pemphigoid is an autoimmune blistering disorder. It results from circulating and tissue-bound autoantibodies directed against bullous pemphigoid antigen one (BPAg1) or bullous pemphigoid antigen two (BPAg2), or both. It usually appears in individuals who are elderly patients as pruritic tense subepidermal blisters on the axilla and groin. Dyshidrosiform bullous pemphigoid is a variant of bullous pemphigoid that typically appears as itchy, potentially hemorrhagic or purpuric, recurrent or persistent blisters on the palms and soles of elderly patients. Typical bullous lesions of bullous pemphigoid may concurrently or subsequently appear on other body sites. The features of patients with dyshidrosiform bullous pemphigoid are reviewed. At least 84 patients with dyshidrosiform bullous pemphigoid have been reported. The condition occurs equally in men and women. Disease onset, for most of the patients, occurred between the ages of 60 years to 94 years. Patients present with blisters on both their palms and soles (67%), just their soles (30%) or just their palms (3%); progression of bullous pemphigoid to other areas of their body occurred in 72% of the patients. Systemic corticosteroids are the mainstay of therapy for dyshidrosiform bullous pemphigoid. Topical corticosteroids, and systemic dapsone or immunosuppressants, may also be necessary to obtain clinical resolution of the blisters. Nearly all of the patients improve with treatment. Neurologic conditions (10 individuals) and psychiatric disorders (two individuals) were discovered in dyshidrosiform bullous pemphigoid patients. Dyshidrosiform bullous pemphigoid should be considered in all elderly individuals who present with the new onset of palmar and plantar blisters that are either recurrent or recalcitrant to therapy—especially if bullae subsequently appear on other areas of the body.

## Data Availability

No new data were created or analyzed in this study. Data sharing is not applicable to this article.
